# Spontaneous pneumothorax as indicator for Birt-Hogg-Dubé syndrome in paediatric patients

**DOI:** 10.1186/1471-2431-14-171

**Published:** 2014-07-03

**Authors:** Paul C Johannesma, Ben EEM van den Borne, Johannes JP Gille, Ad F Nagelkerke, JanHein TM van Waesberghe, Marinus A Paul, R Jeroen A van Moorselaar, Fred H Menko, Pieter E Postmus

**Affiliations:** 1 Department of Pulmonary Diseases, VU University Medical Center, PO Box 7057, 1007, MB Amsterdam, The Netherlands; 2 Department of Pulmonary Diseases, Catharina Hospital, Eindhoven, The Netherlands; 3 Department of Clinical Genetics, VU University Medical Center, Amsterdam, The Netherlands; 4 Department of Paediatrics, VU University Medical Center, Amsterdam, The Netherlands; 5 Department of Radiology, VU University Medical Center, Amsterdam, The Netherlands; 6 Department of Thoracic Surgery, VU University Medical Center, Amsterdam, The Netherlands; 7 Department of Urology, VU University Medical Center, Amsterdam, The Netherlands; 8 Family Cancer Clinic, Antoni van Leeuwenhoek, The Netherlands Cancer Institute, Amsterdam, The Netherlands

**Keywords:** Birt-Hogg-Dubé syndrome, BHD, Folliculin, *FLCN*, Spontaneous pneumothorax, Renal cell cancer, Fibrofolliculomas

## Abstract

**Background:**

Birt-Hogg-Dubé syndrome (BHD) is a rare autosomal dominantly inherited disorder caused by germline mutations in the folliculin (*FLCN*) gene. Clinical manifestations of BHD include skin fibrofolliculomas, renal cell cancer, lung cysts and (recurrent) spontaneous pneumothorax (SP). All clinical manifestations usually present in adults > 20 years of age.

**Case presentations:**

Two non-related patients with (recurrent) pneumothorax starting at age 14 accompanied by multiple basal lung cysts on thoracic CT underwent *FLCN* germline mutation analysis. A pathogenic *FLCN* mutation was found in both patients confirming suspected BHD. The family history was negative for spontaneous pneumothorax in both families.

**Conclusion:**

Although childhood occurrence of SP in BHD is rare, these two cases illustrate that BHD should be considered as cause of SP in children.

## Background

Birt-Hogg-Dubé syndrome (BHD) is a rare tumour predisposition syndrome first described in 1977 [1]. The syndrome is characterized by skin fibrofolliculomas, lung cysts, (recurrent) spontaneous pneumothorax (SP) and renal cell cancer. The underlying cause is a germline mutation in the folliculin (*FLCN*) gene located on chromosome 17p11.2. Clinical manifestation usually appears after the age of 20 years. We here present two cases of BHD with episodes of recurrent pneumothorax of which the first episode occurred at the age of 14 years.

## Case presentation

### Case 1

**Figure 1:**
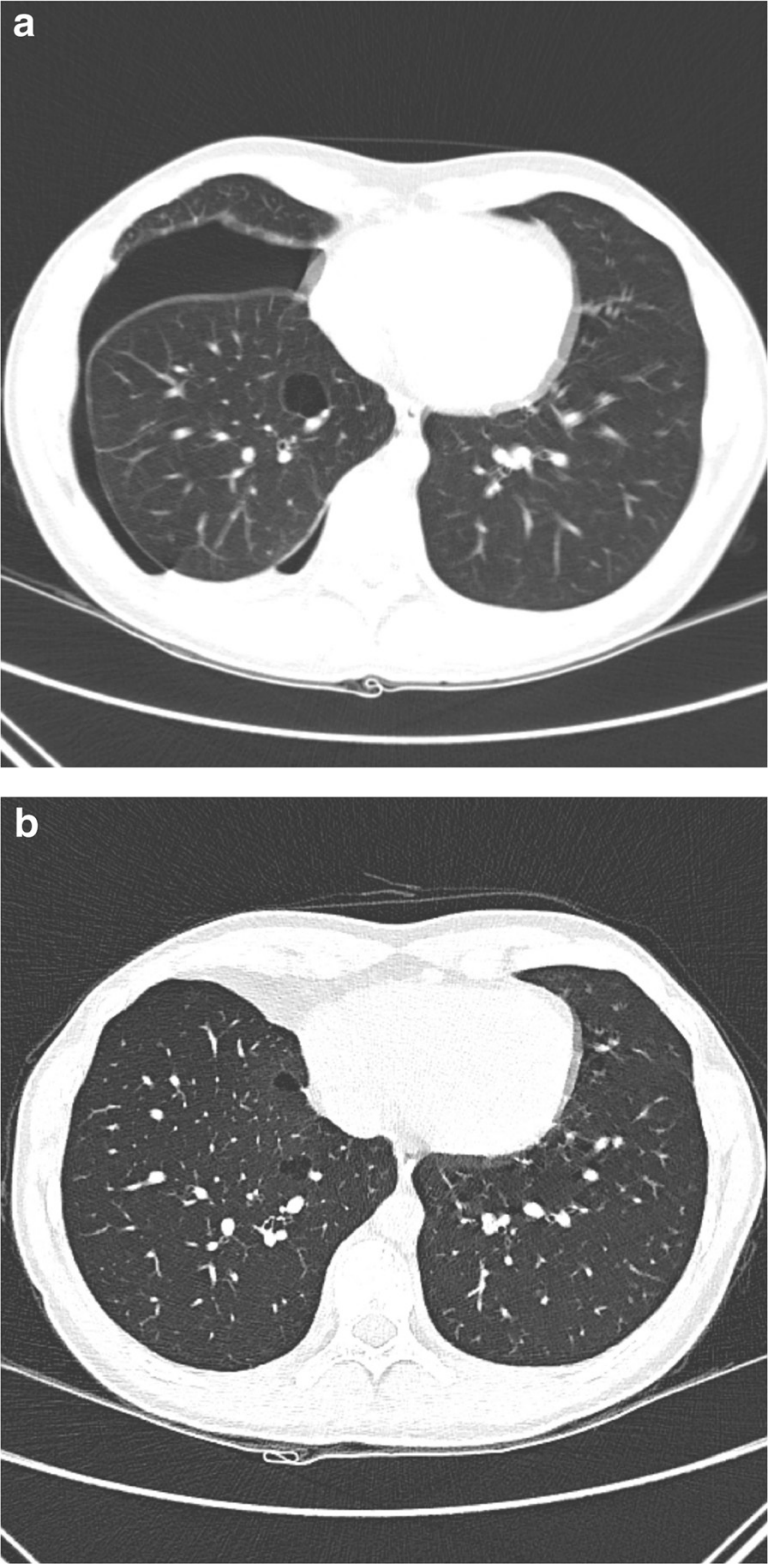
**Thoracic CT of case 1. a.** CT shows a pneumothorax with a intraparenchymal located cyst with small septa. **b.** Shows multiple cysts in the lower lobes (under the carina), in the same patient after treatment of the pneumothorax.

A 14-year-old Caucasian boy, without any medical problems in the past, non-smoker, was admitted to the Emergency Department due to shortness of breath and right-sided chest pain which increased when bending over. In general, he looked healthy and he had no fever. Breath sounds over the right hemithorax were reduced. Routine laboratory tests showed no abnormalities. Chest X-ray showed a right-sided pneumothorax with a complete collapse of the right lung. After drainage by catheter, thoracoscopic pleurodesis was performed. Approximately 8 months later right-sided pneumothorax recurred and was treated by partial right-sided pleurectomy. In the subsequent two years the patient had another two recurrences, both treated by catheter drainage. Because of the recurrent episodes of pneumothorax a CT of his chest was performed, which showed multiple cysts below the level of the carina in the lung parenchyma adjacent to the visceral pleura (subpleurally), predominantly on the right side. (CT-thorax, Figure 1a and b) BHD was suspected and genetic testing confirmed a pathogenic splice-site mutation in the gene (c.1177-5_1177-3delCTC). Skin fibrofolliculomas and renal abnormalities were absent in this patient. Subsequently other family members were counselled by the clinical geneticist. Three family members were also affected. The family history was negative for renal cell cancer (RCC) or (recurrent) episodes of pneumothorax.

### Case 2

**Figure 2:**
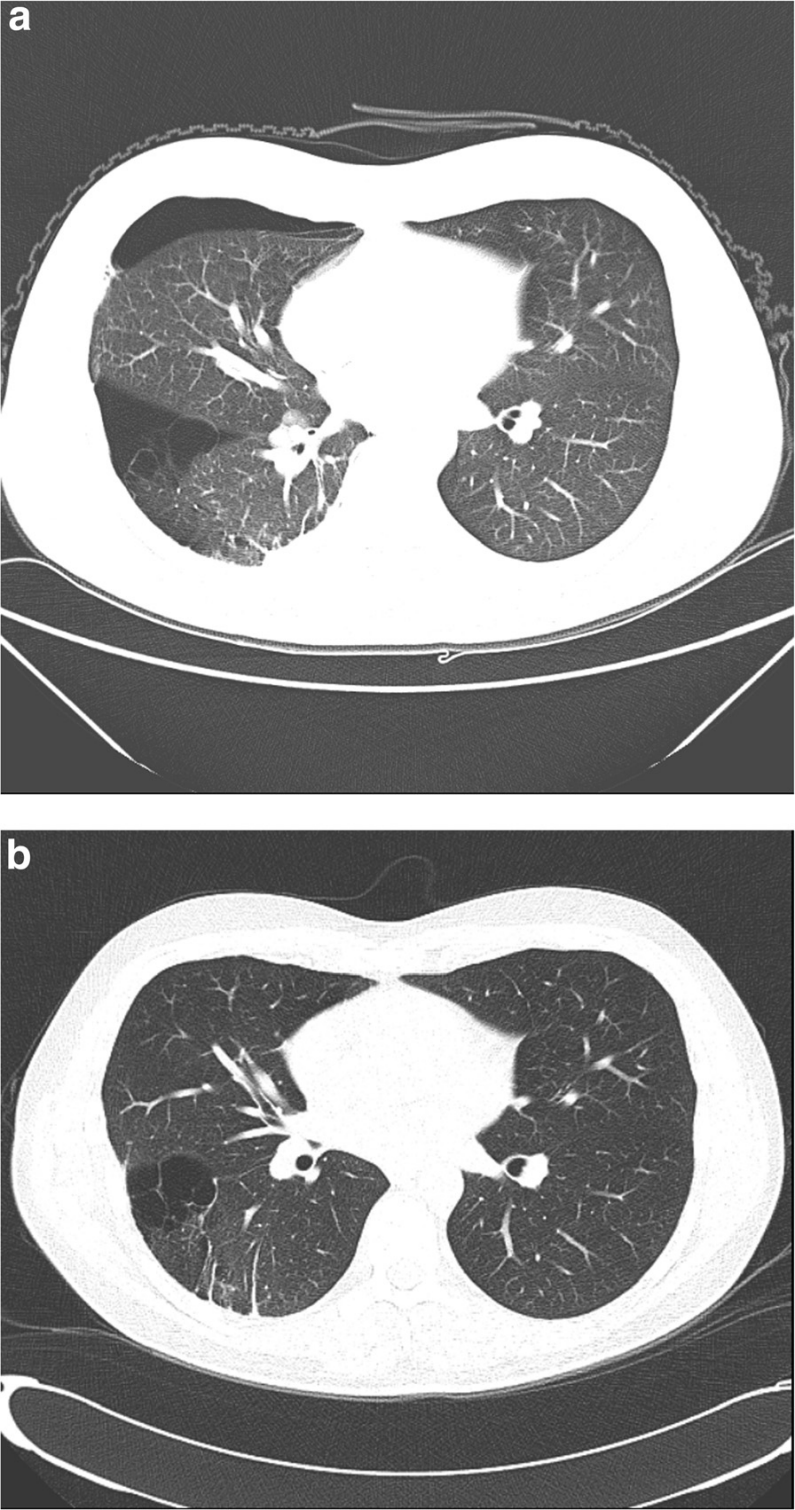
**Thoracic CT of case 2. a.** CT image of pneumothorax with a cluster of lung cysts in the right lower lobe. **b.** Shows a lung cyst located subpleurally in the right lower lobe, abutting the main fissure as shown after resolving of the pneumothorax.

A 19-year-old Caucasian male presented at the Emergency Department due to recurrent right-sided pneumothorax. The first episode had occurred at age 14 and was treated by video-assisted-thoracoscopic surgery (VATS). A chest CT now revealed a completely collapsed right lung with multiple air-filled structures described as bullae at the ventro-cranial side of the right lower lobe. An uncomplicated VATS procedure was performed. Although the thoracic CT showed multiple cysts mainly in the basal parts of the right lung, none were seen at operation. Total pleurectomy was performed. Two years later the patient was referred to our center for evaluation for BHD as this syndrome had been diagnosed in several family members. A chest CT now showed several small cysts in both lungs in the parenchyma and larger cysts resembling ‘bullae’ mainly in the right lung (CT-thorax, Figure 2a and b). Genetic testing confirmed a pathogenic splice-site mutation (c.1301-7_1304del;1323delCinsGA) of the *FLCN* gene., which confirmed the diagnosis of BHD. Skin fibrofolliculomas and renal abnormalities were absent in this patient. Family members had skin fibrofolliculomas, but no RCC or pneumothorax had been observed.

## Conclusions

We report here two paediatric patients with recurrent spontaneous pneumothorax as a manifestation of the Birt-Hogg-Dubé syndrome (BHD). Lung cysts were seen in both patients, predominantly located below the level of the carina, located subpleurally in the lung parenchyma. The location of these cysts is typical for BHD.

In general, the incidence of paediatric primary and secondary SP is approximately 4 per 100.000 males and 1.1. per 100.000 females per year, which is much less common than in adulthood [2,3]. The mean age at presentation of PSP in paediatric cases is between 13.8-15.9 years and occurs commonly in tall, thin males [4]. A specific racial/ethnic predominance has not been described in literature [2]. The underlying cause in spontaneous SP in adulthood is often unknown in the primary form, but secondary SP may be due to an inflammatory or connective tissue disease, infection, malignancy, foreign body aspiration or congenital malformation [5]. Subpleural bullae, mainly in the apex of the lung, are found in 76-100 per cent of adult PSP patients during VATS and thoracotomy [6]. Among non-smokers with a history of PSP, 81 per cent have bullae [7]. The general underlying mechanism of PSP in childhood seems to be connective tissue changes, which predispose to spontaneous leaking of air from the airways into the pleural space. Several case series suggest a relation between subpleural blebs/bullae and the occurrence of SP [2,8]. The incidence of blebs and bullae detected on CT in children with SP is between 45% and 100% [9]. The clinical significance of these blebs and bullae remains unclear [4].

SP in children is often diagnosed based on medical history and physical examination, confirmed by chest radiography. The additional value of chest CT is unclear. In the British Thoracic Society (BTS) guidelines a CT of the chest is indicated when chest radiography is negative of clinical signs for SP or for determining appropriate management strategies. Because blebs, bullae and cysts are only visible on CT, which is performed in a minority of cases, the diagnosis BHD is likely to be missed.

Adult literature reports a recurrence rate in primary SP of approximately 30 percent, with a range of 16 to 52 percent [10]. In children a recurrence rate up to 61 percent has been reported and seems to be higher compared to the adult data [11]. A smaller study by Ouanes-Berbes et al. reported a 19% overall recurrence rate over 7 year follow-up in a young adult cohort, with a 72 percent incidence of bullae [12].

In adults, multiple lung cysts are associated with Birt-Hogg-Dubé syndrome, with a basal rather than apical distribution. The literature on the incidence of BHD syndrome in adults with PSP is very limited. Ren and colleagues found a prevalence of 9.8% in 102 apparently adult SP patients [13]. In a pilot study among 40 apparently primary SP patients we found a pathogenic *FLCN* mutation in 3 (7.5%) patients [14].

Previously, SP has been reported twice in paediatric BHD patients both confirmed by the demonstration of a pathogenic *FLCN*-mutation. The first patient had one episode of SP at age of 7 years. Chest CT was not performed. The second patient at age of 16 years, with a positive family history for SP, had in total 6 episodes of SP on both sides with, multiple basally located cysts on chest CT [15,16]. The youngest BHD patient with pneumothorax we observed was an 18 year old woman with two episodes of right sided spontaneous pneumothorax with lung cysts [17].

BHD [OMIM no. 135150] is a rare autosomal dominant inherited disorder caused by germline mutations in the (*FLCN*) gene located on chromosome 17p11.2. The BHD gene encodes for the protein folliculin which is expressed in skin, kidney and nephrons and type 1 pulmonary alveolar epithelial cells. The function of folliculin has not been fully clarified [18,19]. Clinically BHD is characterized by skin fibrofolliculomas, a 50-fold increased risk for development of (recurrent) SP, multiple lung cysts predominantly in the basal parts of the lung, renal cysts and renal cancer from the age of 20 [17,18]. Patients are advised to undergo lifelong surveillance, preferably by periodic renal MRI or ultrasound. Because the clinical expression can vary and is age-dependent, BHD syndrome in patients who present with pneumothorax cannot be excluded when no renal abnormalities or skin lesions are found [20]. The differential diagnosis of patients with multiple lung cysts include emphysema, cystic bronchiectasis, honeycomb change, cavitated infective nodules, pulmonary Langerhans cell histiocytosis (LCH), lymphangioleiomyomatosis (LAM), lymphocytic interstitial pneumonia (LIP), follicular bronchiolitis, amyloidosis, light chain deposition disease (LCDD) and Birt-Hogg-Dubé (BHD) syndrome [21]. The cystic pattern in BHD differs from that observed in other lung diseases; In BHD multiple thin-walled pulmonary cysts of various sizes are observed, predominately distributed to the lower medial and subpleural regions of the lung with cysts abutting or including the proximal portion of the lower pulmonary veins or arteries [22-24].

Treatment of SP in children does not differ from that in adults and depends on size and underlying cause. Both BTS and American College of Chest Physicians (ACCP) have published different recommendations for treatment of primary SP in adults as well paediatric patients. Multiple studies in adult literature advocate aspiration as a first treatment option in PSP. For secondary SP or recurrent SP the ACCP and BTS recommend tube thoracostomy and surgical recurrence prevention [2,25]. Whether treatment of pneumothorax in BHD patients needs to be more aggressive than in idiopathic SP is still under debate [18].

In conclusion we report here two cases of Birt-Hogg-Dubé syndrome in children with recurrent pneumothorax. As SP in the paediatric population is relatively rare, BHD should be considered as underlying cause, especially when there is a positive family history for pneumothorax, skin fibrofolliculomas or renal cell cancer. BHD patients are at increased risk for renal cell cancer and therefore diagnosis is important for the affected children and their family members. Therefore we suggest that a thorough family history, low dose chest CT (with the restraints of using CT scans in the paediatric population) and easy accessible genetic testing for BHD are important options for paediatric patients with (recurrent) SP should be performed, even when skin manifestation are absent. We also suggest more research is needed on the prevalence of BHD in the pediatric population with a history of (recurrent) SP.

### Consent

Written informed consent was obtained from both patients for publication of this Case report and any accompanying images. Both patients were over the age of 18 when the manuscript was submitted to this journal.

### Ethics

The retrospective data collected for this Case report was part of patient care and was performed in accordance with the Declaration of Helsinki. Therefore exemption was granted by our ethics committee. We obtained written informed consent from both patients for publication of this Case report and any accompanying images. Copy of written consent forms are added as additional supplement.

## Conclusions

As SP in the paediatric population is relatively rare, BHD should be considered as underlying cause, especially when there is a positive family history for pneumothorax, skin fibrofolliculomas or renal cell cancer. BHD patients are at increased risk for renal cell cancer and therefore diagnosis is important for the affected children and their family members. Therefore we suggest that a thorough family history, low dose chest CT (with the restraints of using CT scans in the paediatric population) and easy accessible genetic testing for BHD are important options for paediatric patients with (recurrent) SP should be performed, even when skin manifestation are absent. We also suggest more research is needed on the prevalence of BHD in the pediatric population with a history of (recurrent) SP.

## Abbreviations

BHD: Birt-Hogg-Dubé syndrome; FLCN: Folliculin; SP: Spontaneous pneumothorax; CT: Computed tomography; MRI: Magnetic resonance imaging; VATS: Video assisted thoracoscopic surgery; BTS: British thoracic society; LCH: Langerhans cell histiocytosis; LAM: Lymphangioleiomyomatosis; LIP: Lymphocytic interstitial pneumonia; LCDD: Light chain deposition disease; ACCP: American College of Chest Physicians; PSP: Primary spontaneous pneumothorax.

## Competing interests

All authors have no conflicts of interest to disclose.

## Authors’ contributions

PCJ collected the data, provided a substantial contribution to conceptation, design and drafting/writing of the manuscript, gave final approval of the version to be published and agreed to be accountable for all aspects of the work. BEEMvandenB provided a substantial contribution to the drafting/writing of the manuscript, reviewed and revised it critically, provided the clinical data of patient case 1 as attending pulmonologist, gave final approval of the version to be published and agreed to be accountable for all aspects of the work. JJPG provided the genetic testing and analysis on both patients. Provided a substantial contribution to review and revise the manuscript critically, gave final approval of the version to be published and agreed to be accountable for all aspects of the work. AFN provided a substantial contribution to review and revise the manuscript critically, supervised the analysis and interpretation of the clinical patients data, supervised as attending pediatrician, gave final approval of the version to be published and agreed to be accountable for all aspects of the work. JHTMvanW, provided a substantial contribution to review and revise the manuscript critically, provided and interpreted the thoracic imaging of both patients as attending radiologist, gave final approval of the version to be published and agreed to be accountable for all aspects of the work. MAP, provided a substantial contribution to review and revise the manuscript critically, provided the clinical data of patient case 1 and 2 as attending thoracic surgeon, gave final approval of the version to be published and agreed to be accountable for all aspects of the work. RJAvanM, provided a substantial contribution to review and revise the manuscript critically, gave final approval of the version to be published and agreed to be accountable for all aspects of the work. FHM, provided a substantial contribution to review and revise the manuscript critically provided the genetic testing on Folliculin of patient case 1 and 2, contributed as the attending clinical geneticist of both families, gave final approval of the version to be published and agreed to be accountable for all aspects of the work. PEP, provided a substantial contribution to conceptation, design and drafting/writing, contributed to review and revise the manuscript critically, contributed as attending pulmonologist of patient case 2, gave final approval of the version to be published and agreed to be accountable for all aspects of the work.

## Pre-publication history

The pre-publication history for this paper can be accessed here:

http://www.biomedcentral.com/1471-2431/14/171/prepub
